# BAGE: a Bayesian framework for age prediction based on PBMC gene expression data

**DOI:** 10.1186/s12859-026-06519-8

**Published:** 2026-06-13

**Authors:** Veronica Suaste, Maria L. Daza-Torres, J. Cricelio Montesinos-López, Hilde Loge Nilsen

**Affiliations:** 1 Department of Biosciences, University of Oslo, Oslo, Norway; 2 Department of Public Health Sciences, University of California, Davids, CA USA; 3 Department of Microbiology, Oslo University Hospital, Oslo, Norway; 4 CRESCO - Centre for Embryology and Healthy Development, University of Oslo, Oslo, Norway; 5 Instituto de Matemáticas, Universidad Nacional Autónoma de México, Cuernavaca, Mexico

**Keywords:** Age, Prediction, Bayesian, Transcriptome, Aging, PBMC

## Abstract

**Background:**

Estimating chronological age from biological data is increasingly important for clinical and research studies of aging and age related diseases. In regards to transcriptomic data, it has been proven that chronological age prediction widely depends on sample type. While single-cell RNA-seq based clocks using PBMC single-cell data have been reported, to our knowledge no publicly available bulk RNA-seq age predictor has been trained specifically on peripheral blood mononuclear cells (PBMCs). To address this gap, we aggregated 16 publicly available PBMCs bulk transcriptomic datasets comprising 174 healthy individuals (ages 4–81 years, sex balanced) and implement BAGE (Bayesian framework for age prediction from gene expression data). BAGE implements a Bayesian linear mixed model that incorporates dataset as a random intercept to capture batch effects and technical heterogeneity.

**Results:**

In this study we evaluated multiple BAGE implementations under the leave-one-out cross validation strategy. The evaluated models vary in age parametrization, counts transformation and variable selection approaches. Best model performance achieved a coefficient of determination ($R^2$) of 0.86 and mean absolute error (MAE) of 5.5, outperforming elastic net model and RNAAgeCalc tool on our PBMC data. This performance was achieved with square root parametrization of age and the resulting predictors constitute a concise consensus signature of 70 genes, stable across datasets. Via over-representation analysis the signature points to biological pathways related to natural killer cells.

**Conclusions:**

Explicitly modeling study-level heterogeneity and using a signature specific to PBMC improved predictive accuracy relative to an elastic net base-line and the RNAAgeCalc multi-tissue calculator. The BAGE framework is adaptable to larger, heterogeneous cohorts and is readily extensible for integration with additional omics layers. Subject to external and longitudinal validation, the selected gene set could provide interpretable biomarkers of immune aging.

## Introduction

Aging is a process characterized by a gradual decline in physiological function and an increased risk of chronic diseases, including cardiovascular diseases and neurodegenerative disorders, cancer, and arthritis [1–3]. Chronological age is an important risk factor for these conditions and remains a strong predictor of overall mortality. As a consequence, accurate estimation of chronological age based on clinical tests or biological patterns has become a critical step in both clinical and research contexts, where it can provide an essential baseline for the study of aging and age-related diseases.

Importantly, the manifestations of aging vary considerably between individuals, even when matched by chronological age [4, 5]. This heterogeneity has been attributed to differences in underlying biological processes that led to the concept of biological age [6]. Measurement of biological age has the potential to improve our understanding of aging mechanisms and to enable clinical applications, such as assessing disease risk and tracking the response to healthy aging interventions. However, any effort to quantify biological age requires an accurate chronological age prediction as a reference point, underscoring the importance of developing robust models for age estimation.

Epigenetic clocks, based on DNA methylation data, have achieved remarkable predictive accuracy of chronological age [7, 8] and have been widely adopted in aging research, particularly the second generation of methylation clocks that target aging phenotypes, disease, and mortality risk [9, 10]. However, despite their predictive power, epigenetic clocks are limited in their ability to reveal mechanistic links between the epigenome and aging processes [11, 12]. Consequently, multi-omic approaches and tissue-specific models have been argued to be necessary to extract mechanistic insights in addition to maintaining an accurate age estimation [11, 13].

Transcriptomic models have emerged alongside methylation clocks as a complementary strategy [14–17]. Predicting chronological age from gene expression has the advantage that gene expression more directly reflects the current functional state of a cell [18], which facilitates interpretation and supports modeling of dynamic changes in cell physiology across age. Transcriptome-based prediction models have been developed in multiple species and sources of biological material, offering opportunities to also investigate evolutionary conservation in aging trajectories and to identify shared or divergent molecular mechanisms [14, 15, 19].

Transcriptomic age prediction models are commonly developed using machine learning approaches [14–16, 20], with elastic net regression representing the most implemented framework. Its methodological strength lies in the integration of penalization and variable selection, which enables effective modeling of high-dimensional gene expression data [21].

The transcriptomic clocks to date illustrate both considerable potential and methodological heterogeneity. Peters et al. [17] used whole-blood microarray’s profiles to derive a transcriptomic age measure: they report 1,497 age-associated genes but built their predictor using the 11,908 genes common across cohorts, applied a leave-one-cohort-out training scheme, and obtained a mean absolute error (MAE) of 7.8 years. Jung et al. [16] combined pathway priors with a generalized linear model on multi-tissue RNA-seq data, selecting 818 genes and reporting an MAE of 8.2 years and $R^2=0.75$ in 10-fold cross-validation. Ren et al. [14] developed RNAAgeCalc from 9,448 RNA-seq samples spanning 26 tissue types, identifying 1,618 common age-related genes and providing tissue-specific calculators; when evaluated on blood-related tissue (blood vessel), their best model (using 15,516 genes) produced a Pearson correlation of 0.84 and MAE of 5.29 years. Other studies focus on single tissues: for example, an ensemble of LDA classifiers applied to 133 skin fibroblast RNA-seq samples achieved MAE = 7.7 years, median absolute error of 4.0 years and $R^2=0.81$ under leave-one-out cross-validation.

Some of these studies also support the well established observation that aging signatures are dependent on tissue [14, 22–24]. Although several transcriptomic predictors are trained on multiple tissues and include whole blood or blood-vessel profiles [14, 17], and recent work has proposed single-cell RNA-seq based clocks developed on PBMC single-cell data [25, 26], to our knowledge, no predictor has been developed specifically for bulk RNA-seq data from peripheral blood mononuclear cells (PBMCs). Single-cell transcriptomic clocks provide mechanistic insight into cell aging at the cost of greater complexity and sequencing depth, whereas bulk transcriptomic clocks integrate signals across cell populations and are generally more practical and cost-effective for large cohorts. Therefore, we focused on building and evaluating a PBMC-targeted bulk-transcriptome predictor to address this gap.

Although whole-blood sampling minimizes processing steps, PBMC isolation selectively removes red blood cells and granulocytes, leaving lymphocytes (T, B, NK cells) and monocytes. This cellular enrichment facilitates the study of adaptive and innate immune responses [27, 28]. Additionally, PBMCs have been suggested to be an intact source of biomarkers, in contrast to whole blood, where biomarker detection remains challenging due to the large amount of non-specific background [29]. Moreover, PBMCs are amenable to a range of downstream functional analyzes and may be the preferred material to biobank where sample volumes are limited, e.g. from newborn or children.

In this work, we present BAGE (Bayesian framework for age prediction from gene expression data) used in PBMC transcriptomic data to predict chronological age. We used 16 publicly available datasets comprising 174 healthy individuals, spanning an age range of 4 to 81 years and with balanced sex representation along the aggregated sample. We hypothesize that the relationship between gene expression and age is broadly consistent across cohorts and that dataset-specific systematic shifts in expression magnitude can be captured by a random intercept. In contrast to common strategies that remove batch effects during preprocessing, our framework directly models specific variations in the dataset using random effects. This approach enables the model to account for technical heterogeneity while preserving shared biological signals across cohorts. To implement this, we fitted a Bayesian linear mixed model with the dataset as a random intercept. This modeling framework is particularly appropriate for high-dimensional genomic settings where the number of predictors often exceeds the number of samples (*p>>n*), a situation that can lead to overfitting, multicollinearity, and identifiability problems [30, 31].

We benchmarked performance of the model against elastic net and transcriptomic clock RNAAgeCalc [14]. In addition, we examined predictors for their potential as biomarkers of biological aging or as members of pathways associated with aging.

## Material and methods

### Data collection

We manually searched PubMed for studies using the query (transcriptomic OR transcriptome OR RNA-seq) AND (PBMC OR Peripheral Blood Mononuclear cells). For each returned article, we inspected the methods and supplementary material, excluded single-cell studies, and retained only those reporting bulk RNA-seq with publicly available data (i.e., a GEO/SRA accession). As a second filtering step, we confirmed that age and sex were available for every sample, either in the study metadata or explicitly in the publication. Applying these criteria produced 16 datasets comprising 174 healthy samples, which were used in our research. A complete list of datasets, accession numbers, and source publications is available in Supplementary Table S1.

### RNA-seq data processing

We obtained raw sequencing reads from publicly available data in the Sequencing Reads Archive (SRA) [32]. We downloaded all reads from each dataset through SRA toolkit (v3.0.3). The metadata about the samples were manually downloaded using the SRA Run Selector when applicable, otherwise the metadata were mined directly from the article involved, particularly gender and age.

We pre-processed raw reads with FastQC [33] (v0.11.9) for quality assessment. In order to have homogeneous quality for all datasets, we used trimming and quality filtering methods from fastp [34] (v0.23.2), we used a minimum base quality score of 30 and an average quality score threshold of 30 for the whole read. We also used –cut_right and –overrepresentation_analysis parameters to filter-out all sequences from adapter contamination. In the case where the sample library type was paired we also used the parameter –correction to correct mismatched base pairs in overlapped regions of paired ends.

After processing the reads, we align them with the human genome reference GRCh38, ensembl release 109 [35], using STAR aligner [36] (v2.7.10b). With the same aligner, using the –quantMode GeneCouns parameter, we generated the gene-level counts. STAR requires a known library strandedness to select the appropriate gene-level counts output. Several source publications did not report strandedness, so we performed an additional alignment with Salmon (v1.9.0) using –libType A to let Salmon infer library type (and strandedness) automatically.

In further analysis, we excluded genes with low count (*<5*) for more than 75% of the samples.

### Data transformation

#### Counts transformation

We performed counts normalization in order to account for technical variability across samples, such as library size, sequencing depth, and/or gene length:

**Regularized logarithm (rlog):** We transformed raw counts using the DESeq2 regularized log (rlog) method [37]. The rlog outputs data on $log_2$ scale and incorporates normalization for library size while stabilizing variance across expression levels. The transformation was executed with parameter blind = TRUE to avoid using the information of the sample during variance estimation.

**Log**$_{10}$ **transformation:** We converted raw counts to pseudo-counts by adding 1 to each value and then applied a $log_{10}$ transformation, i.e. we used $log_{10}(count+1)$. Although this approach is simple, it often improves predictive performance by compressing differences that span orders of magnitude. We interpret the large original differences as largely driven by variable sequencing depth and therefore consider this transformation a pragmatic way to stabilize the input for our models.

**Transcript per Million (TPM)**: TPM normalization scales counts by both gene length and sequencing depth, enabling comparison of expression levels across genes and samples. We computed TPM as follows: $\text {TPM}_i = \frac{\text {N}_i/\text {l}_i}{\sum _{j}(\text {N}_j/\text {l}_j)} \times 10^6$, where ${N}_i$ is the read count for gene *i* and ${l}_i$ is the length for gene *i*. The read length we used is given by the non-overlapping exons of all isoforms. This gene length was extracted, using gtftools [38] v0.9.0, from the same human genome reference annotations used in the alignment step; see above 2.2.

**Counts per Million (CPM): ** CPM normalization adjusts for sequencing depth by scaling each gene count to the total library size. We computed the CPM as follows $\text {CPM}_{i,k} = \frac{N_i}{T_k} \times 10^6$ where ${N}_i$ is the read count for gene *i* and $T_k$ is the total reads mapped to sample *k*.

For differential gene expression analysis, raw counts were used as required by DESeq2 [37].

#### Age transformation

**Square-root** ($\sqrt{age}$): It has been suggested that gene expression and age relationship should be examined beyond linear models [39, 40]. Following this recommendation, we ran a Box–Cox grid search over *λ * (see Supplementary Figure S7). Motivated by these results, we modeled the response variable (age) either on the raw scale (*λ = 1*) or after a square-root transformation (*λ = 0.5*), which provided a useful and interpretable nonlinear option in our data.

### Model

We model chronological age as a function of both fixed and random effects within a Bayesian linear mixed model:

1 $$\begin{aligned} \textbf{Y} = \textbf{1}\mu + \textbf{X}\boldsymbol{\beta } + \textbf{Z}\textbf{v} + \mathbf {X_g}\boldsymbol{\beta _g} + \boldsymbol{\epsilon }, \end{aligned}$$

where $\textbf{Y}$ is the *(n× 1)* vector of observed chronological ages for *n* individuals, $\textbf{1}$ is the *(n× 1)* vector of ones, *μ * is the intercept, $\textbf{X}$ is the *(n× k)* design matrix for fixed effects (e.g., sex, ethnicity/ancestry, and clinical covariates such as smoking or alcohol use), $\boldsymbol{\beta } = (\beta _1, \ldots , \beta _k)^\top$ is the *(k× 1)* vector of fixed-effect coefficients, $\textbf{v}$ is the *(q× 1)* vector of dataset-specific random effects, $\textbf{Z}$ is the *(n× q)* design matrix associated with these random effects, $\boldsymbol{\beta _g}$ is the *(p× 1)* vector of genetic random effects, $\mathbf {X_g}$ is the *(n× p)* standardized gene expression matrix (rows represent individuals and columns represent genes), and $\boldsymbol{\epsilon }$ is the *(n× 1)* vector of random errors.

We assume a flat prior for the intercept (*μ *), i.e., $f(\mu ) \propto 1$, which is approximately specified as $\mu \sim N(0, \sigma ^2_0)$ with a large variance $\sigma ^2_0 = 10^{10}$. The remaining priors are defined as follows: $\boldsymbol{\beta } \mid \sigma _{\beta }^2 \sim N(0, \sigma _{\beta }^2 \textbf{I}_k), \quad \textbf{v} \mid \sigma _q^2 \sim N(0, \sigma _q^2 \textbf{I}_q), \quad \boldsymbol{\beta _g} \mid \sigma _{\beta _g}^2 \sim N(0, \sigma _{\beta _g}^2 \textbf{I}_p), \quad \boldsymbol{\epsilon } \mid \sigma ^2 \sim N(\textbf{0}, \sigma ^2 \textbf{I}_n),$ where $\textbf{I}_m$ denotes the *(m× m)* identity matrix.

For the variance components, we assign scaled inverse chi-squared priors:

$$ \sigma _{\beta }^2 \sim \chi ^{-2}(v_\beta , S_\beta ), \quad \sigma _q^2 \sim \chi ^{-2}(v_q, S_q), \quad \sigma _{\beta _g}^2 \sim \chi ^{-2}(v_{\beta _g}, S_{\beta _g}), \quad \sigma ^2 \sim \chi ^{-2}(v, S), $$

where $\chi ^{-2}(v,S)$ denotes the scaled inverse chi-squared distribution with shape parameter *v* and scale parameter *S*.

In genomic prediction, the number of genes (*p*) used to predict chronological age is typically much larger than the number of individuals (*n*) in the sample (*p ≫ n*). This high dimensionality can introduce computational challenges when exploring the posterior distribution of the regression coefficients $\boldsymbol{\beta _g}$. When the main goal is prediction, a common strategy is to reduce the dimensionality of the problem by directly simulating values of $\textbf{g} = \mathbf {X_g}\boldsymbol{\beta _g}$ (the genetic effects) instead of sampling $\boldsymbol{\beta _g}$ itself [41].

Since $\boldsymbol{\beta _g} \mid \sigma _{\beta _g}^2 \sim N(0, \sigma _{\beta _g}^2 \textbf{I}_p)$, this induces a prior for $\textbf{g}$ given by:

$$ \textbf{g} \mid \sigma _{\beta _g}^2 \sim N(0, \sigma _{\beta _g}^2 \mathbf {X_g}\mathbf {X_g}^\top ) = N(0, \sigma _g^2 \textbf{G}), $$

where $\sigma _g^2 = p\sigma _{\beta _g}^2$ and $\textbf{G} = \frac{1}{p}\mathbf {X_g}\mathbf {X_g}^\top$ is a linear kernel known as genomic relationship matrix [42].

Under this parameterization, the model in Equation 1 becomes:

2 $$\begin{aligned} \textbf{Y} = \textbf{1}\mu + \textbf{X}\boldsymbol{\beta } + \textbf{Z}\textbf{v} + \textbf{g} + \boldsymbol{\epsilon }, \end{aligned}$$

with induced priors $\textbf{g} \mid \sigma _g^2 \sim N(0, \sigma _g^2 \textbf{G})$ and $\sigma _g^2 \sim \chi ^{-2}(v_g, S_g)$, where $v_g = v_{\beta _g}$ and $S_g = pS_{\beta _g}$.

Model 2 is commonly referred to as the Bayesian Genomic Best Linear Unbiased Predictor (GBLUP) model [41]. The implementation was carried out in the R statistical software [43] using the BGLR package developed by Pérez and de Los Campos [31]. In all analyses, we used the default hyperparameter settings of the BGLR package. Specifically, BGLR assigns scaled inverse chi-squared priors to all variance components, with default degrees of freedom $v_\beta = v_q = v_{\beta _g} = v = 5$. The scale parameters $S_\beta$, $S_q$, $S_{\beta _g}$, and *S* are set so that the mode of the prior distributions for $\sigma _{\beta }^2$, $\sigma _q^2$, $\sigma _{\beta _g}^2$, and $\sigma ^2$ corresponds to a predefined proportion of the total phenotypic variance (the sample variance of the response, see Appendix 2 in [41] for details). In practice, this procedure allocates a specified share of the total variance of the phenotypes to the different components of the model.

We denote the proposed Bayesian framework and its implementation as BAGE. The model includes biological sex as a fixed effect covariate to control for systematic sex-specific variation. Additional covariates, such as ethnicity/ancestry, clinical information (e.g., smoking and drinking habits), and other known factors that influence aging, can also be included as fixed or random effects when available. Including this additional metadata may improve prediction accuracy and enable more reliable conclusions across diverse populations.

Dataset membership is treated as a random intercept, allowing the model to capture batch effects and technical heterogeneity. Gene expression counts, following appropriate transformation, are incorporated as random effects to accommodate the natural heterogeneity and correlation structure among genomic features. This formulation assumes a consistent relationship between gene expression and age across cohorts while accounting for systematic shifts in expression magnitude specific to each dataset.

### Variable selection

We evaluated two variable-selection methods and compared their effects on model performance.

**DESeq2-assisted selection:** We used the DESeq2 R package [37] to fit a differential expression model for each gene with age or transformed age as the primary variable. To account for technical variation across studies and known sex differences in aging, we included dataset and gender as covariates in the model. We ranked genes by adjusted p-value and selected the top 2000 genes; we denote this set as the DESeq-assisted variables.

**Univariate linear-model selection (manually selected):** For each gene, we fitted a simple linear model using lm (R stats) with age or transformed age as the response variable, transformed counts (one of $log_{10}$, TPM, CPM or rlog) as the predictors and gender and dataset as a covariates. We collected the p-values from these fits, applied Benjamini–Hochberg correction for multiple testing, and selected the top 2000 genes using adjusted p-value. We refer to this collection as the manually selected variables.

We combined a straightforward univariate linear model, which permits flexible count transformations and is model agnostic, with the well established DESeq2 framework that explicitly models count distributions and accounts for covariates. Using both approaches provided complementary perspectives on age associated genes and increased confidence that selected features reflect biological signal rather than method specific artifacts. Importantly, both approaches are compatible with our strategy of modeling dataset effects as a random intercept within the BAGE framework.

Variable selection was performed independently within each training–testing split to avoid information leakage

### Model evaluation

Several versions of the BAGE model (Equation 2) were implemented to predict chronological age using PBMC transcriptome data. These model variants differ in three main aspects: (i) the normalization applied to gene expression counts (regularized rlog, $\log _{10}$ transformation, TPM or CPM), (ii) whether the response variable (age) was modeled on the raw scale or after applying a square-root transformation ($\sqrt{\text {age}}$), and (iii) the method used for variable selection (manual selection vs. DESeq2-assisted selection). All models presented below incorporate biological sex as a fixed effect in the predictor structure, while treating the dataset and gene expression counts (with their respective transformations) as random effects.

Models **M1–M8** correspond to formulations using manual variable selection. Models M1-M4 predict raw chronological age using $\log _{10}$-transformed counts, rlog-transformed counts, TPM or CPM, respectively. Models M5–M8 extend these formulations by modeling $\sqrt{\text {age}}$ instead of raw age, allowing the model to capture potential non-linear relationships between age and gene expression.

Similarly, models **D1–D8** employ DESeq2-assisted variable selection. Models D1–D4 predict raw age using either $\log _{10}$-transformed counts, rlog-transformed counts, TPM or CPM, respectively. Models D5 and D8 mirror these structures, but use square-root transformed age as response variable.

Together, these model variants allow us to systematically evaluate the impact of predictor normalization, response transformation, and variable-selection strategy on the predictive performance of the BAGE framework for estimating chronological age.

We used a leave-one-out cross validation approach for the model’s performance assessment. Each sample in the dataset was used as a test and all remaining samples as a training set. The same split was used for both variable selection and model fitting.

We also evaluated the model performance as a function of the number of selected variables (Methods 2.5): for each leave-one-out cross-validation iteration we fit each model with ranked gene sets of sizes ranging from 50 up to 2000, with increments of 50 (50, 100,150,...,2000).

We adopted the combined set of evaluation metrics reported in previous transcriptomic age prediction studies to ensure consistency and comparability [14–17]. Specifically, performance of the model was assessed using the coefficient of determination ($R^2$), Pearson’s correlation coefficient (*ρ *), root mean squared error (RMSE), mean absolute error (MAE), and the median absolute deviation of residuals (MAD). Let *n* be the number of samples, $y_i$ and ${\hat{y}}_i$ the true and predicted age of sample *i* correspondingly. Then: $R^2 = 1 - {\sum (y_i - {\hat{y}}_i)^2} / {\sum (y_i - {\bar{y}})^2}$, $\textrm{MAE} = \frac{1}{n}\sum |y_i - {\hat{y}}_i|$, $\textrm{RMSE} = \sqrt{\frac{1}{n}\sum (y_i - {\hat{y}}_i)^2}$, $\textrm{MAD} = \textrm{median}(|y_i - {\hat{y}}_i|)$, and $\rho = \frac{\sum _{i=1}^n (y_i - {\bar{y}})({\hat{y}}_i - \overline{{\hat{y}}})}{\sqrt{\sum _{i=1}^n (y_i - {\bar{y}})^2}\sqrt{\sum _{i=1}^n ({\hat{y}}_i - \overline{{\hat{y}}})^2}}$.

### Validation with disease samples

We collected the disease counterpart samples (N=248) from same studies that provided the training data. In other words, where a study included both healthy and disease participants, only the healthy samples were used to train and evaluate all BAGE models and the matched disease samples were held out and used as an additional validation set. Therefore the disease validation is independent at the sample level but not at the study level, which can reduce the effective independence of the test set because study specific technical or biological characteristics could be shared. We processed disease RNA-seq data with the same read pre-processing pipeline (trimming and quality filtering) and used same reference genome for alignment and gene quantification (Methods 2.2). Nevertheless, these steps were executed separately for each condition.

After evaluation of all versions of the BAGE model (Equation 2 ) we selected the best one (see “Variable selection and model performance” section) and used it to predict age to disease samples. In terms of implementation, we ran model M5 with the BAGE_PBMC signature using BGLR package [31]. Disease samples were held out with respect to the response. The dataset variable was included in the model (as a random intercept / design matrix column) so that study-specific baseline effects were estimated from the healthy training data and applied when predicting the held-out disease samples.

### Model comparison

**Elastic Net:** We implemented an elastic net model using the glmnet R package (v 4.1-9) [44]. We set the mixing parameter alpha = 0.5 to balance L1 and L2 penalties. For each train-test split, we tuned the regularization strength lambda with cross-validation using cv.glmnet method with default lambda sequence (nlambda=100, lambda.min.ratio=0.01), and then fitted the final model with glmnet function using the selected lambda. For fair comparison, we forced the model to include the dataset and gender as categorical variables, setting penalty.factor parameter equal to zero for these specific variables. We evaluated predictive performance using leave-one-out cross-validation where each sample served once as the test set and the remaining samples as the training set. The complete workflow variable selection, lambda tuning, model fitting and testing was executed independently for each count-normalization method and age parametrization to ensure an unbiased comparison.

**RNAAgeCal:** We applied the RNAAgeCalc age-prediction tool [14] to our samples. For input we provided raw counts (exprtype = “count”) and specified gene length given by the non-overlapping exons of all isoforms. This gene length was extracted, using gtftools [38] v0.9.0, from the same human genome reference annotations used in the alignment step. We set stype = “all” so that the pre-trained clock used samples from all races. For tissue specification, we evaluated three options; two that most closely match PBMC: “blood”, “blood vessel”, and the one that includes all tissues (“all_tissues”). For the signature argument, we ran all available alternatives and report performance for each signature–tissue combination.

### Enrichment and network analysis

Protein–protein interaction (PPI) network and functional enrichment analyzes were performed using the STRING web tool (version 12; https://string-db.org) [45]. The selected genes were queried against a background of 13,169 of genes expressed after low count filtering. Interactions were retrieved based on evidence from experiments, databases, co-expression and text mining. The minimum required interaction score was set to 0.4 for medium confidence. The resulting network was visualized and analyzed to identify clusters using the integrated Markov clustering (MCL) algorithm option.

Functional enrichment for Gene Ontology (GO), KEGG pathways and WikiPathways was carried out using the integrated STRING enrichment module, applying a false discovery rate (FDR) correction for multiple testing (significance threshold: FDR * < 0.05*). The enrichment results were summarized and visualized based on adjusted p-values and signal scores.

A schematic overview of the complete analytical workflow, including data integration, preprocessing, variable selection, and model evaluation, is provided in Supplementary Figure S1.

## Results

We assembled a cohort of 174 RNA-seq samples derived from peripheral blood mononuclear cells (PBMCs) of healthy individuals, as outlined in Methods 2.1. These samples were distributed across 16 publicly available datasets from the Sequencing Reads Archive (SRA), which are detailed in the SupplementaryTable S1. Each dataset originates from independent studies in the literature that primarily investigated comparisons between healthy controls and diseased or treated individuals, from which we selected only healthy controls in all cases. Given that different studies utilized varying protocols for RNA extraction, library preparation and sequencing, notable batch effects were observed that we modeled as a random effect in the model, see Model 2.4.

**Fig. 1:**
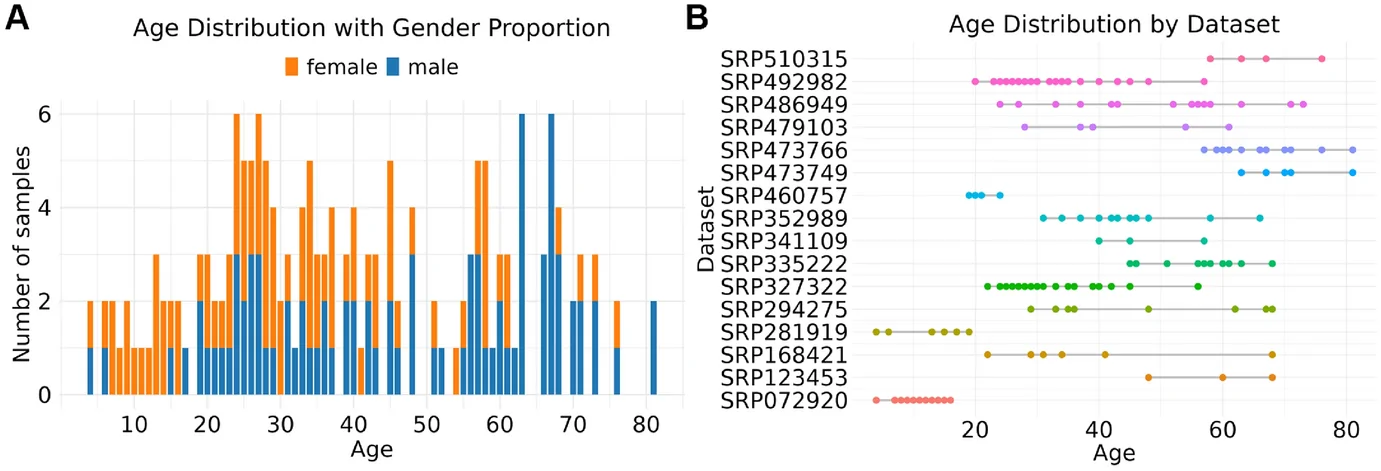
Samples distribution. **A** Samples age distribution by gender, *y* axis represent the number of sample stacked, *x* axis age in years. **B** Samples distribution by age and dataset, each dot represent a sample and color represent the dataset indicated in *y* axis as well, *x* axis age in years

The sample cohort is balanced with respect to gender, with 88 males and 86 females contributing to the dataset. The samples also span a broad age range, from 4 to 81 years, as visualized in Fig. 1A. This wide age distribution is essential for the objectives of the present study. While individual studies did not uniformly cover the full age spectrum, the combined dataset achieves broad coverage: each decade of age is represented by a minimum of two independent dastsets, supporting analyzes across age groups despite uneven ranges per study, Fig. 1B.

**Table 1:** Model prediction performance evaluated using Coefficient of Determination ($R^2$), Root Mean Squared Error (RMSE), Mean Absolute Error (MAE), Median Absolute Deviation (MAD) of residuals, and Pearson Correlation (*ρ *). The number of selected genes is also reported for each model. For each metric, the best performing model among those with manual variable selection is highlighted in bold, and the best performing model among those with DESeq-assisted variable selection is highlighted in bold italics.

Model	# genes	${R^2}$	${\rho }$	MAE	MAD	RMSE
M1 = Manual + log10(counts)	150	0.845	0.919	5.900	4.871	7.676
M2 = Manual + rlog(counts)	700	0.823	0.907	6.024	4.502	8.186
M3 = Manual + TPM(counts)	400	0.833	0.913	5.852	4.653	7.942
M4 = Manual + CPM(counts)	450	0.828	0.910	5.986	4.585	8.060
M5 = M1 + sqrt(age)	150	**0.861**	**0.928**	**5.520**	3.995	**7.273**
M6 = M2 + sqrt(age)	200	0.843	0.919	5.845	4.393	7.712
M7 = M3 + sqrt(age)	350	0.837	0.915	5.741	4.123	7.850
M8 = M4 + sqrt(age)	350	0.845	0.920	5.528	**3.733**	7.644
D1 = DESeq2 + log10(counts)	50	0.822	0.907	6.386	5.237	8.211
D2 = DESeq2 + rlog(counts)	250	0.817	0.904	6.505	5.408	8.331
D3 = DESeq2 + TPM(counts)	250	0.806	0.898	6.725	5.430	8.567
D4 = DESeq2 + CPM(counts)	250	0.808	0.899	6.651	5.202	8.527
D5 = D1 + sqrt(age)	150	***0.832***	***0.912***	***6.011***	***4.442***	***7.989***
D6 = D2 + sqrt(age)	200	0.827	0.909	6.248	4.626	8.111
D7 = D3 + sqrt(age)	150	0.813	0.902	6.418	4.739	8.416
D8 = D4 + sqrt(age)	450	0.815	0.903	6.283	4.447	8.371

### Variable selection and model performance

We evaluated the performance of our BAGE models, M1–M8 and D1–D8, (Methods 2.6) using a leave-one-out cross-validation approach. In this setup, one sample is left out as the test set, while the remaining samples are used to train the model. This procedure is repeated iteratively, ensuring that each sample is used once as a test instance, which allows for a assessment of the model’s generalization and overall performance. It is important to note that in each iteration, the same test-training split is applied consistently to both the variable selection step and the model fitting phase.

We employed two distinct strategies for variable selection; see details in Methods 2.5. For both strategies, we evaluated model performance as a function of the number of selected genes: for each leave-one-out (LOO) cross-validation iteration, we ran each model with ranked gene sets of sizes ranging from 50 up to 2000, with increments of 50.

The leave-one-out cross-validation performance for all models is summarized in Table 1. The performance metrics reported correspond to the optimal number of genes that yielded the highest coefficient of determination ($R^2$), prioritizing a smaller number of genes where the performance was similar. For models using manually selected genes, M5 (which uses $\log _{10}$-transformed counts and square-root transformed age) achieved the best performance. It attained the highest $R^2$ (0.861) and Pearson correlation (0.928), together with the lowest error rates(MAE: 5.52, and RMSE: 7.273) using only 150 genes. In terms of MAD, model M8 (which uses CPM-transformed counts and square-root–transformed age) achieved a sliest better numbers of 3.733 vs 3.995.

Moreover, consistent results were observed for models based on DESeq2-selected genes. Here, model D5 (which also uses $\log _{10}$-transformed counts and square-root–transformed age) performed the best among its group, achieving superior metrics ($R^2 = 0.832$, *ρ = 0.912*) compared to D1, D2, and D4.

**Fig. 2:**
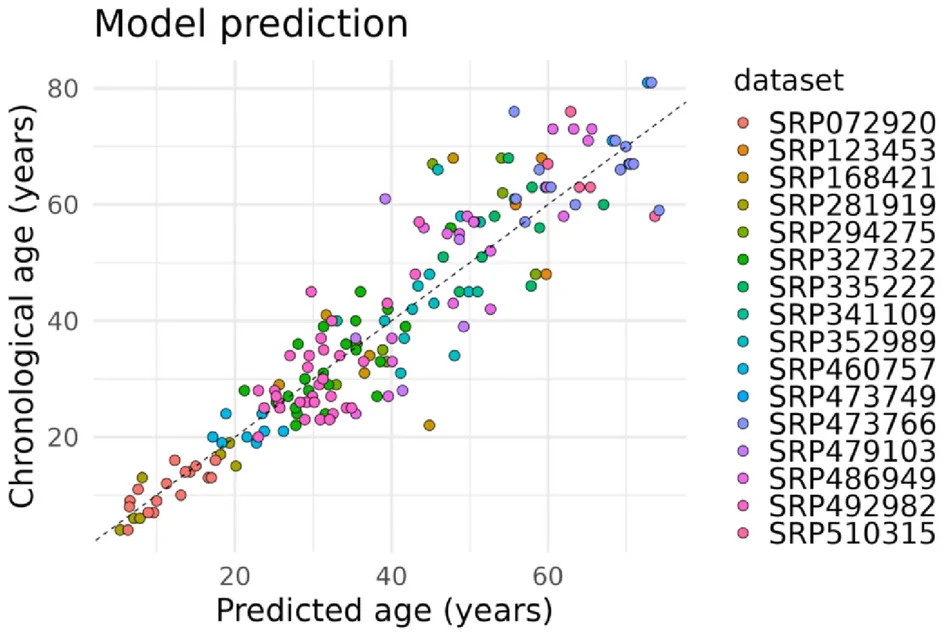
Model M5 predictions of chronological age, with the leave-one-out cross-validation strategy. The plot compares predicted versus chronological age, with each point representing an individual. Points are colored by dataset to illustrate dataset-specific effects. The dashed line indicates the 1:1 relationship, where perfect prediction would lie

In both variable selection strategies, models that incorporated $\sqrt{\text {age}}$ as the response variable (M5-M8, D5-D8) consistently outperformed those modeling raw age. Furthermore, the manually selected gene set generally led to better predictive accuracy than the DESeq2-selected set, with model M5 demonstrating the strongest performance in all configurations. The relationship between predicted and chronological age, with the leave-one-out cross-validation strategy, for the top-performing model M5 is shown in Fig. 2.

### Comparison with standard models

To benchmark the performance of the best BAGE model (M5), we compared it with two established approaches: an Elastic Net regression model and the published multi-tissue clock, RNAAgeCalc [14].

The elastic-net model was evaluated using leave-one-out cross-validation and the same count transformations and age parameterizations that we used for our models. The performance of all the correspondent models is reported in the Supplementary Table S2, for comparison, we selected the best performance ($R^2=0.811$), achieved with *rlog*-transformation counts and square-root parametrization of age, reported in Table 2.

RNAAgeCalc, provided as a ready-to-use tool with its own internal normalization and published gene coefficients, was applied directly to the raw count data using the tool’s recommended input format [14]. RNAAgeCalc allows for the specification of sample type (tissue) and alternative signatures (sets of genes) configurations, enabling tailored predictions; see more details on methods and the original publication [14]. The complete performance results are given in the Supplementary Table S4. For comparative clarity, we emphasize the two RNAAgeCalc settings that yielded the strongest performance according to different criteria: the “blood” tissue with the “GTExAge” signature (highest $R^2$) and the “blood” tissue with the “Peter” signature (highest Pearson correlation). Therefore, these two configurations are reported alongside our models as the most favorable RNAAgeCalc baselines.

The comparative results are summarized in Table 2. Model M5, a BAGE model with $\sqrt{\text {age}}$, outperformed both benchmark methods across all performance metrics, achieving a higher $R^2$ and lower error rates (MAE, MAD, RMSE) than the Elastic Net model and substantially outperforming RNAAgeCalc.

**Table 2:** Performance comparison. Age prediction performance ($R^2$, *ρ *, MAE, MAD, RMSE) for M5 (BAGE), Elastic Net, and RNAAgeCalc, with the number of genes or signatures indicated. For elastic net the number of genes correspond to the median of all leave-one-out iterations

Model	# genes	${R^2}$	${\rho }$	MAE	MAD	RMSE
M5 (BAGE)	150	**0.861**	**0.928**	**5.520**	**3.995**	**7.273**
Elastic Net	40	0.811	0.901	6.547	4.838	8.454
RNAAgeCalc + Peters	289	− 0.306	0.803	18.826	17.527	22.260
RNAAgeCalc + GTExAge	312	0.453	0.753	12.053	11.057	14.403

### Sensitivity and stability analysis in variable selection

To evaluate whether selected genes are more meaningful than random selection, we used the best-performing model (M5) and compared its performance when fitted with genes selected via our manual method versus genes selected at random.

**Fig. 3:**
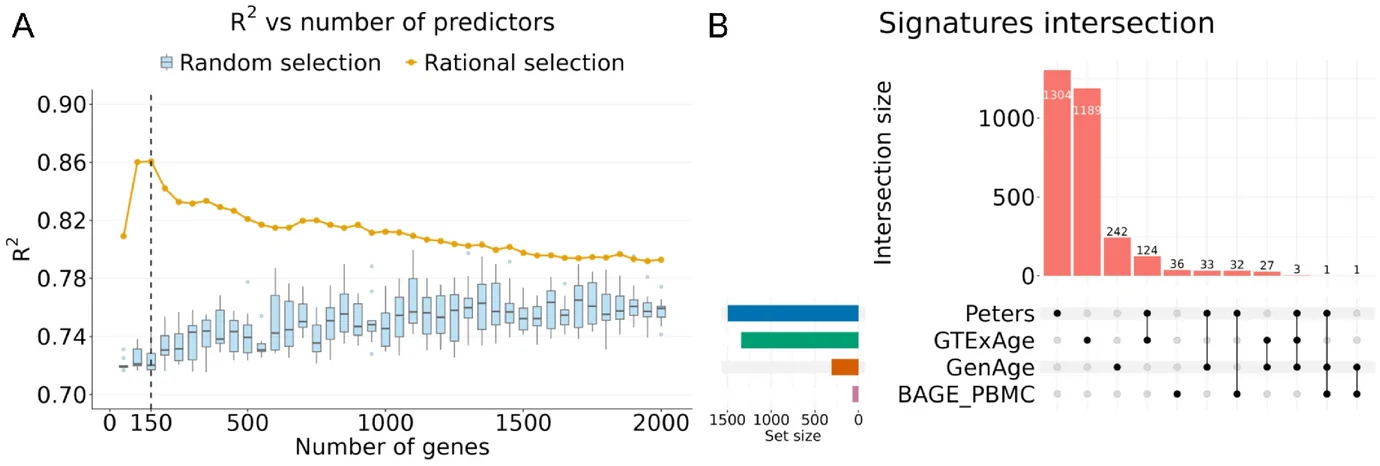
**A** Model M5 performance using randomly selected gene sets (50–2000 genes, increments of 50), with boxplots showing $R^2$ distributions across 10 replicates and line showing Model M5 performance with manually selected genes ranked by adjusted p-value, showing $R^2$ in the test set versus number of genes. Note that the best prediction performance is reached with 100–150 genes. **B** Intersection between established signatures and our signature (BAGE_PBMC). Horizontal bars denote the size in number of genes of each one of the signatures, Peters, GTExAge, GenAge, BAGE_PBMC. Vertical bars and the explicit number denote the size of individual or intersection sets among two or more signatures defined by the interconnected dots underneath the panel

For the random selection, we repeatedly sampled gene sets of sizes ranging from 50 to 2000 (in increments of 50), fitting the M5 model 10 times for each set size. Figure 3A presents the resulting $R^2$ values as a function of the number of genes that were randomly selected in the training and testing sets.

These results were compared against the performance of the M5 model when using manually selected genes, which were prioritized by their adjusted p-value from the pre-fitting step to ensure the most relevant genes were selected first for model fitting. The corresponding $R^2$ values for the test set are plotted in Fig. 3B as a function of the number of genes selected.

The performance trajectory for manually selected genes consistently surpasses the cloud of results from random selection across nearly all gene set sizes in the testing set. Furthermore, the manual selection curve exhibits a distinctive peak at 150 genes, confirming the optimal number identified in Table 1, after which the performance plateaus.

Additionally, we evaluated the robustness of feature selection across cross-validation folds for both of our rational selection methods (Section 2.5). Using the univariate linear-model approach, 70 genes were selected in 100% of the folds; with the DESeq2-based method, 76 genes were selected in all folds. Visualizations of the number of genes most frequently selected as well as the intersection between both methods are provided in the Supplementary Figure S3.

We further assessed variable selection stability by (i) subsampling the training set and (ii) bootstrap resampling within leave-one-out folds (see Supplementary Methods, Section S2). In the subsampling experiment, the intersection percentage of the selected genes and our BAGE_PBMC signature increased monotonically in terms of the median from 37% with 50% of the samples to 92% with 90% of the samples (Supplementary Figure S5). In the bootstrap analysis, we collected the top 150 selected genes from each replicate, aggregated these lists across B = 1000 bootstraps per fold, and ranked the genes by their selection frequency. The top 150 genes by overall frequency included 64 genes from the BAGE_PBMC signature (91% of the 70-gene signature; Supplementary Figure S6A). Selection frequencies for this core set were also consistent across leave-one-out folds (Supplementary Figure S6B).

To compare our selected features with previously published aging signatures, we took the intersection of the 150 genes retained in each leave-one-out fold to form a conservative consensus of 70 genes (from 13,169 genes tested after low-count filtering), we refer to this set as BAGE_PBMC signature. The full list of genes is provided in Supplementary File 1. Figure 3B shows the overlap between this BAGE_PBMC set of genes and three signatures from the literature: Peters (1,497 genes) [17], GTExAge (1,343 genes) [14], and GeneAge (307 genes) [46]. Thirty-four of our 70 consensus genes (49%) overlap with at least one published signature; 32 genes overlap with Peters et al., consistent with the use of whole-blood samples in that study, while smaller overlaps are observed with GTExAge and GeneAge. Notably, IGFBP3 is the one gene corresponding to the intersection of our signature BAGE_PBMC, Peters and GeneAge while the one intersecting only BAGE_PBMC and GenAge is PDGFB.

As an external robustness test, we used the BAGE_PBMC signature and our best model M5 (see Methods 2.7) for age prediction to the disease counterpart samples from same studies that provided the training data. When using the disease samples cohort (N=248) as an external validation set, with the caveat that these are not healthy controls and represent different disease contexts, the model produced an aggregated $R^2=0.77$ for chronological age prediction. The complete performance metrics and the predicted versus observed age plot are provided in the Supplementary Table S3 and the Supplementary Figure S2.

**Fig. 4:**
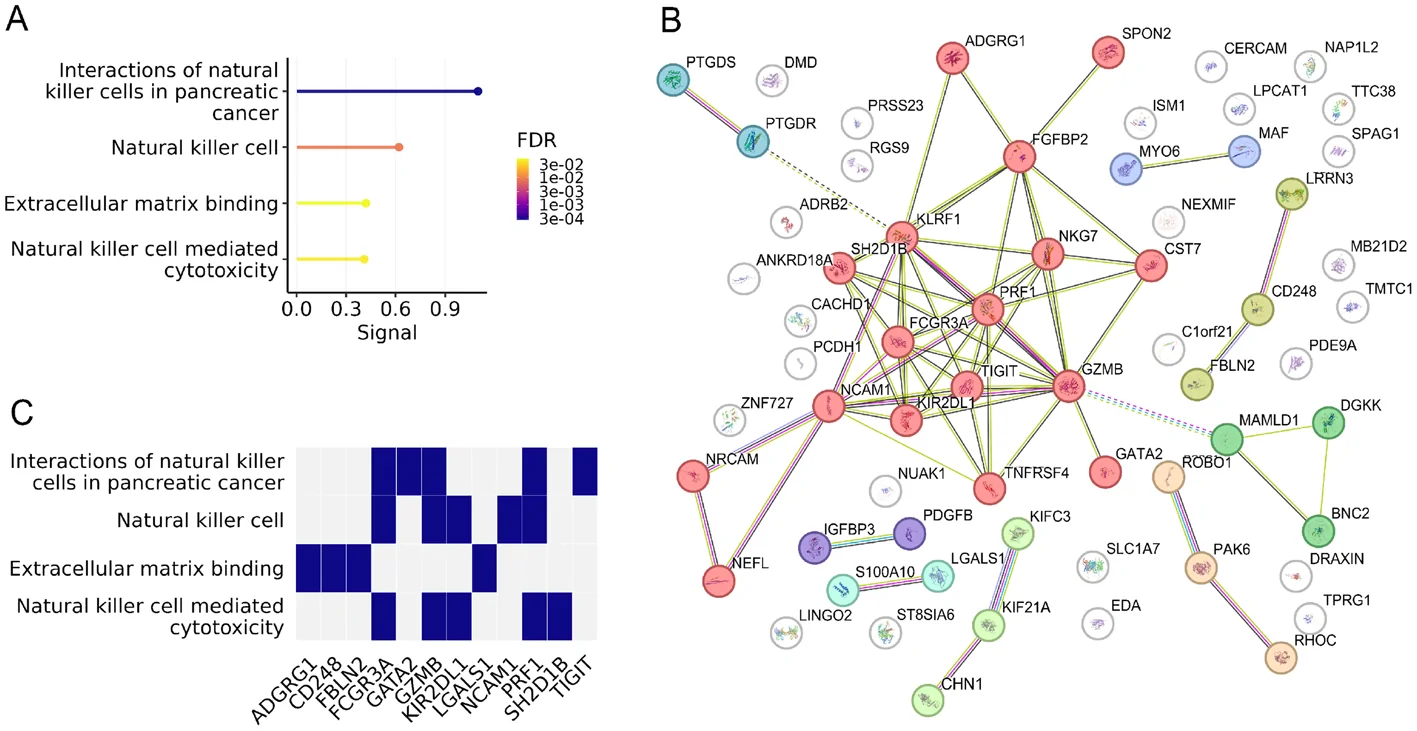
Functional enrichment and network organization of the BAGE_PBMC signature (70 genes). **A** Top over-represented pathways for consensus gene set (set = 70 genes, background=13,169 expressed genes), **B** Co-association network reconstructed from the 70 consensus genes. Node color indicates module membership as defined by Markov clustering (MCL). Largest MCL module (n = 17) is enriched for NK-cell pathways; a secondary module (containing ROBO1, PAK6, RHDC) is enriched for “Activation of RAC1”. **C** Mapping of individual genes in the signature to enriched pathways

To assess the biological coherence of this BAGE_PBMC signature, the set of genes was also subjected to over-representation analysis (Methods 2.9). The top enriched terms (Fig. 4A; full results in Supplementary File 2) included "Interaction of natural killer cells in pancreatic cancer" (FDR = 2.*7 × 10*-4, signal = 1.10) and "Natural killer cell" (FDR = 8.*5 × 10*-3, signal = 0.62).

Complementing enrichment, we reconstructed a co-association/protein– protein interaction network from consensus features. The network showed strong protein–protein interaction enrichment (p = 1.*0 × 10*-16), indicating that consensus genes are biologically more connected than expected by chance (Fig. 4B). We partitioned the network using Markov clustering (MCL) to identify modules: the largest cluster (17 genes) was enriched for NK-cell pathways, while a second cluster (including ROBO1, PAK6, RHDC) was enriched for “Activation of RAC1” (see Supplementary File 3 for full cluster annotations). Finally, we mapped individual signature genes to the enriched pathways and identified PRF1 as one of the key contributors to the NK pathway signal (Fig. reffig:biologyspsrelevanceC).

## Discussion

In our work, we found that a BAGE model applied to PBMC transcriptomes, with the dataset variable modeled as a random effect, outperformed elastic net, a commonly used benchmark. We also observed that the predictors developed for whole blood do not necessarily transfer to PBMCs: RNAAgeCal [14] performed worse in our PBMC samples, reinforcing that aging signatures depend on the type of sample. The genes prioritized by our model pointed to pathways related to Natural Killer (NK) cell as important components of the PBMC aging signature and individual genes were corroborated in the literature as markers of immunosenescence. Finally, we showed that the relationship between age and gene expression was better captured by a non-linear parametrization: prediction performance improved when we modeled age after applying a square-root transformation.

### BAGE framework and its potential

The superior performance of the BAGE model indicates that explicitly modeling cross-study shifts via random effects, coupled with Bayesian regularization, is an effective strategy for multi-study prediction settings, especially when *p>> n*. It has been suggested that gene expression and the relationship with aging should be explored beyond linear models [39, 40]. In line with this recommendation, our analyzes demonstrate superior predictive performance when chronological age is modeled as square-root compared with a purely linear term. This phenomenon was consistent across expression transformation and variable selection strategies. This observation suggests that the pattern captured by our model follows a common non-linear trajectory in which the transcriptional change is relatively more pronounced at younger ages and decelerates with advancing age; the square-root parametrization effectively accommodates this early acceleration followed by later flattening.

Our findings highlight the potential of random-effects frameworks for integrating multiple transcriptomic datasets. By accounting for variability specific to each study, the model learns from systematic differences across cohorts while preserving shared biological signals. By explicitly modeling these sources of variation rather than treating them as noise, the approach enhances generalization across diverse populations and experimental conditions. This strategy therefore supports the development of more compact and robust aging clocks that are likely to maintain predictive accuracy when applied to external validation datasets.

Our external test on disease samples achieving an aggregated correlation coefficient $R^2$ of 0.77 provides an illustrative check of generalization, but must be interpreted with caution. Disease status can introduce systematic transcriptomic shifts that differ from healthy variation and therefore can bias age predictions. Because our goal was to establish a calibrated baseline for chronological age, we only interpret the result on disease samples as a pragmatic generalizability check rather than evidence that the model captures biological age in disease contexts. Further research is required to assess the utility of BAGE for clinical application; independent healthy cohorts, matched longitudinal data, and dedicated studies that distinguish specific disease effects from aging.

### Age prediction comparison

The application of RNAAgeCalc to our PBMC data produced a lower performance than our M5 model even with their highest observed metric(*ρ = 0.80* vs *ρ = 0.92*). Comparison of multiple metrics is important: low $R^2$ can still produce a high correlation that indicates that the trend is captured shifted by an intercept, so metric choice influences interpretation. Nevertheless, the consistently poorer performance of RNAAgeCalc in all metrics (Table 2) is most aptly attributed to sample type specificity, PBMCs and whole blood exhibit different gene-expression’s profiles [47], further supporting the view that transcriptional aging signatures do not generalize uniformly across sample types.

Elastic-net regression remains a strong and commonly used baseline for transcriptomic clocks [14, 19] and attains good performance in our data ($R^2 = 0.811$). However, the Bayesian mixed-model framework (BAGE) paired with a conservative and rational feature selection strategy delivered superior performance ($R^2 = 0.861$).

Together, these results underscore two points. First, explicitly modeling study-level heterogeneity and employing Bayesian regularization can improve prediction and generalizability in multi-study settings. Second, because transcriptional aging signatures vary by sample or tissue type and cohort [14, 24], it is essential to evaluate multiple methodological paradigms and transparently report their implementations. Different algorithms and selection strategies, depending on the sample type or population, may yield distinct, yet complementary biological insights without sacrificing statistical rigor.

### Gene predictors and their biological relevance

The emergence of NK cell-related pathways within our PBMC age signature is consistent with increasing evidence that NK cells contribute to age-associated neuroinflammation and play critical roles in immune surveillance of senescent cells [48–50]. Functionally NK cells act as first responders of the immune system, eliminating pre-malignant cells, as well as cells infected with viruses or other intracellular pathogens via cytotoxic mechanisms [51, 52]. In our results, the “NK cell mediated cytotoxicity” pathway was highlighted as correlated with the PBMC aging signature; notably, PRF1 (perforin), a gene implicated in NK cytotoxic function, is part of our signature (Fig. 4) and previous studies have linked age dependent declines in NK cytotoxicity to reduced expression of perforin or impaired perforin release [53, 54] .

However, mechanistic links between NK cells and aging remain incompletely resolved. The literature is inconsistent on whether NK cell counts change with age: some studies report increasing NK numbers, while others report no systematic change [52]. In recent work, specific molecular changes reported to correlate with age in PBMC were tested in samples spaning from 10 to 79 years and found no correlation between cell type proportions and chronological age [55]. Similarly, the literature on the cytotoxic potential of NK across lifespan is inconsistent [52]. Our computational association between NK pathways and chronological age therefore aligns with a biologically plausible role for NK cells, but does not by itself resolve these conflicting observations. To resolve the ambiguity, follow-up studies should combine functional NK cytotoxicity assays and longitudinal profiling to determine whether observed transcriptional changes reflect true functional decline, compensatory responses, or cohort-specific phenomena. Such a targeted validation would clarify whether NK-related transcriptional changes can serve as robust biomarkers of immunological aging or as mechanistic entry points for interventions.

Importantly, several of the genes we prioritized have independent support as immunosenescence markers (e.g., LRRN3, TIGIT) [56–58], or have been also found in other aging signatures (Fig. 3B, Supplementary File 1), strengthening confidence of the predictors. The largest intersection(N=32 genes) given with the Peters signature is enriched for natural killer cell related pathways when subjected to over representation analysis on that subset (Supplementary Figure S4), indicating that the immune signals we detect have been observed previously. The intersection analysis, reported in results, also highlights IGFBP3 and PDGFB as anchors with potential biological interpretation. The proteins encoded by these genes are linked to pathways related to ageing [59–61]. IGFBP3, which is the only gene shared across our signature, Peters and GenAge signatures, has been linked to cellular senescence and age related pathways: it is the principal binding protein for IGF-1 and thus modulates IGF signaling, it has been reported to up regulate PI3K/Akt/mTOR signaling and to down regulate autophagy during cell aging and it can induce senescence through telomerase suppression in experimental systems [59, 60]. These diverse connections position IGFBP3 as a plausible mediator of age associated cellular programs. Platelet-derived growth factor (PDGFB) has been shown to provoke a complex response that includes both senescence and cellular transformation in a p53 dependent manner in primary fibroblast models [61]. Together, these observations are consistent with our pathway enrichment for immune and senescence related processes and suggest that the PBMC signature captures aspects of immune remodeling and cellular aging biology. Nevertheless, we emphasize caution on the interpretations as bulk cross sectional PBMC data cannot disentangle changes in cell composition from cell regulatory changes and do not establish causality. Targeted validation in sorted cell populations, single cell profiles and functional experiments will be required to test these hypotheses.

### Broader implications

By introducing a BAGE for transcriptomic age prediction, we expect this modeling framework to be readily adopted in broader settings because of its flexibility to accommodate diverse covariates as fixed or random effects. In practice, this means that we can incorporate additional sample characteristics such as ancestry/ethnicity, tissue of origin, clinical covariates or measured cell-type proportions directly into the model to explicitly account for their influence on expression and age prediction. This adaptability should facilitate the application of the method to larger and more heterogeneous cohorts and to multi-study or multi-omic integrations.

Multi-omics approaches are an active and promising direction in aging research [39, 62, 63] and therefore in age prediction. Integrating DNA methylation and gene expression for age prediction, for example, could potentially improve accuracy and increase interpretability: methylation contributes stable, high-accuracy signals, while transcriptomics provides information on the functional state of the cell that aids mechanistic interpretation. Extending BAGE to integrate both or more modalities is technically feasible and could enhance both prediction and biological insight.

From a broader perspective, parallel efforts to map aging signatures across tissues and diverse populations (sex, ethnicity) are essential to build a comprehensive mechanistic understanding of human aging; we anticipate that targeted, tissue-specific investigations will ultimately converge to reveal shared and tissue-specific aging pathways. At the same time, the identification of robust tissue-relevant biomarkers that can track the therapeutic response or aging phenotypes has substantial clinical and societal relevance. The modeling flexibility and interpretability offered by BAGE support both trajectories: it facilitates the synthesis of heterogeneous cohort data toward mechanistic insight and produces candidate marker sets that can be prioritized for validation in longitudinal studies or intervention trials.

### Limitations and future work

A natural limitation when integrating heterogeneous datasets from multiple sources is that we limited gene selection to those that were consistently measured in all studies. Genes absent from at least one dataset for reasons such as limited sequencing depth or read-length–dependent capture are excluded, so our analysis focuses on the intersection of measurable genes. This approach maximizes comparability but likely omits portions of the aging signal that are not consistently captured. We adopted this conservative strategy because a zero count cannot be interpreted unambiguously as a biologically significant lack of expression versus a technical failure to detect the transcript.

Modeling the dataset effect as a random intercept is an appropriate strategy to account for batch effects, as it assumes that differences between datasets are primarily reflected in their baseline levels rather than in their age-related slopes. In other words, while the relationship between gene expression and age is expected to be consistent across datasets, the random intercept captures systematic shifts in magnitude attributable to batch or laboratory effects. However, a limitation of our data is the lack of uniform age coverage across datasets. This incomplete representation may introduce confounding, since effects specific to dataset can inadvertently embed information related to age. Despite this, our main objective was to optimize predictive performance within the constraints of the available data while appropriately modeling batch effects. The results presented here should be interpreted in that context. Future analyzes using datasets with optimally balanced age distributions will allow a more rigorous assessment of generalizability of the model. As large-scale genomic resources continue to expand, we expect that improved age coverage will mitigate this limitation. Notably, the proposed model remains robust and conceptually transferable to settings with more uniform data coverage.

Although our prioritized features show promise as markers of aging, their utility as indicators of biological age remains to be established through external validation. One pragmatic approach is to evaluate the predictor in cohorts affected by accelerated-aging phenotypes versus appropriately matched control groups. Demonstrating a consistent elevation of transcriptomic age or age acceleration in such cases would provide important evidence that the predictor indexes biologically relevant aging processes beyond chronological aging.

Separately, we assessed the stability of the PBMC signature under resampling. Although a stable core set of genes remains reproducibly selected, our subsampling experiments indicate that selection stability improves with larger training sample sizes. These results underline the importance of larger and more diverse cohorts for consolidating the full signature and for translating it into robust biomarkers.

In future work, our framework could accommodate richer nonlinear aging patterns beyond the square-root transformation used in this study. Specifically, a promising direction is to cluster genes with similar nonlinear profiles, as in [40], and then apply Reproducing Kernel Hilbert Spaces (RKHS) regression [41] within each gene cluster. RKHS regression is already implemented within the BGLR package [31], used in our BAGE framework.

## Conclusions

In this study, we developed a Bayesian framework for age prediction from gene expression data (BAGE) focused on PBMC samples. The best model performance was obtained with a non-linear age parametrization ($\sqrt{age}$) achieving $R^2 = 0.86$ and *MAE = 5.5* years. Explicitly modeling study-level heterogeneity and using a signature specific to the sample type improved predictive accuracy relative to an elastic net baseline and the RNAAgeCalc multi-tissue calculator. Interpretation of the BAGE_PMBC signature (70 genes) implicates Natural Killer cell-related pathways providing specific testable mechanistic hypotheses and nominating clear follow-up targets. Key limitations include uneven age coverage in the aggregated datasets. The next priority steps are replication of the predictor in independent and demographically representative PBMC cohorts, and longitudinal or interventional studies to assess sensitivity to biological aging and response to treatment. With successful external validation, BAGE could provide a robust and interpretable transcriptomic signature to monitor immune aging and evaluate therapeutic impact. The framework is adaptable to larger, heterogeneous cohorts and readily extensible to integrate additional omics layers.

## Supplementary Information

Below is the link to the electronic supplementary material.

Supplementary file 3.

Supplementary file 2.

Supplementary file 1.

Supplementary file 4.

## Data Availability

The original datasets analyzed in this research are publicly available and a complete list of accession numbers and source publications is available in the Supplementary Table A1. The source code for analysis and plotting as well as curated data is available on GitHub at https://github.com/vsuaste/BAGE.

## Abbreviations

PBMC: Peripheral blood mononuclear cells
MAE: Mean absolute error
SRA: Sequencing read archive
RMSE: Root mean squared error
MAD: Median absolute deviation of residuals
FDR: False discovery rate
MCL: Markov clustering
LOO: Leave-one-out
