# Supplementary material for: BAGE: a Bayesian framework for age prediction based on PBMC gene expression data
Source: BMC Bioinformatics. 2026 Jun 13;27:193. doi: 10.1186/s12859-026-06519-8 (PMC13505173; doi:10.1186/s12859-026-06519-8)
Supplement: Supplementary file 1 [file 12859_2026_6519_MOESM4_ESM.pdf]

Supplementary Material for: BAGE: A Bayesian framework for age prediction based on PBMC gene expression data

Veronica Suaste[1,3,4*], Maria L. Daza-Torres[2,5†], J. Cricelio Montesinos-López[2†], Hilde Loge Nilsen[3,4]

[1*]Department of Biosciences, University of Oslo, Oslo, Norway.
[2]Department of Public Health Sciences, University of California, Davids, California, USA.
[3]Department of Microbiology, Oslo University Hospital, Oslo, Norway.
[4]CRESCO - Centre for Embryology and Healthy Development, University of Oslo, Oslo, Norway.
[5]Instituto de Matemáticas, Universidad Nacional Autónoma de México, Cuernavaca, México.

*Corresponding author(s). E-mail(s): veronsua@uio.no;
†These authors contributed equally to this work.

S1 Supplementary material

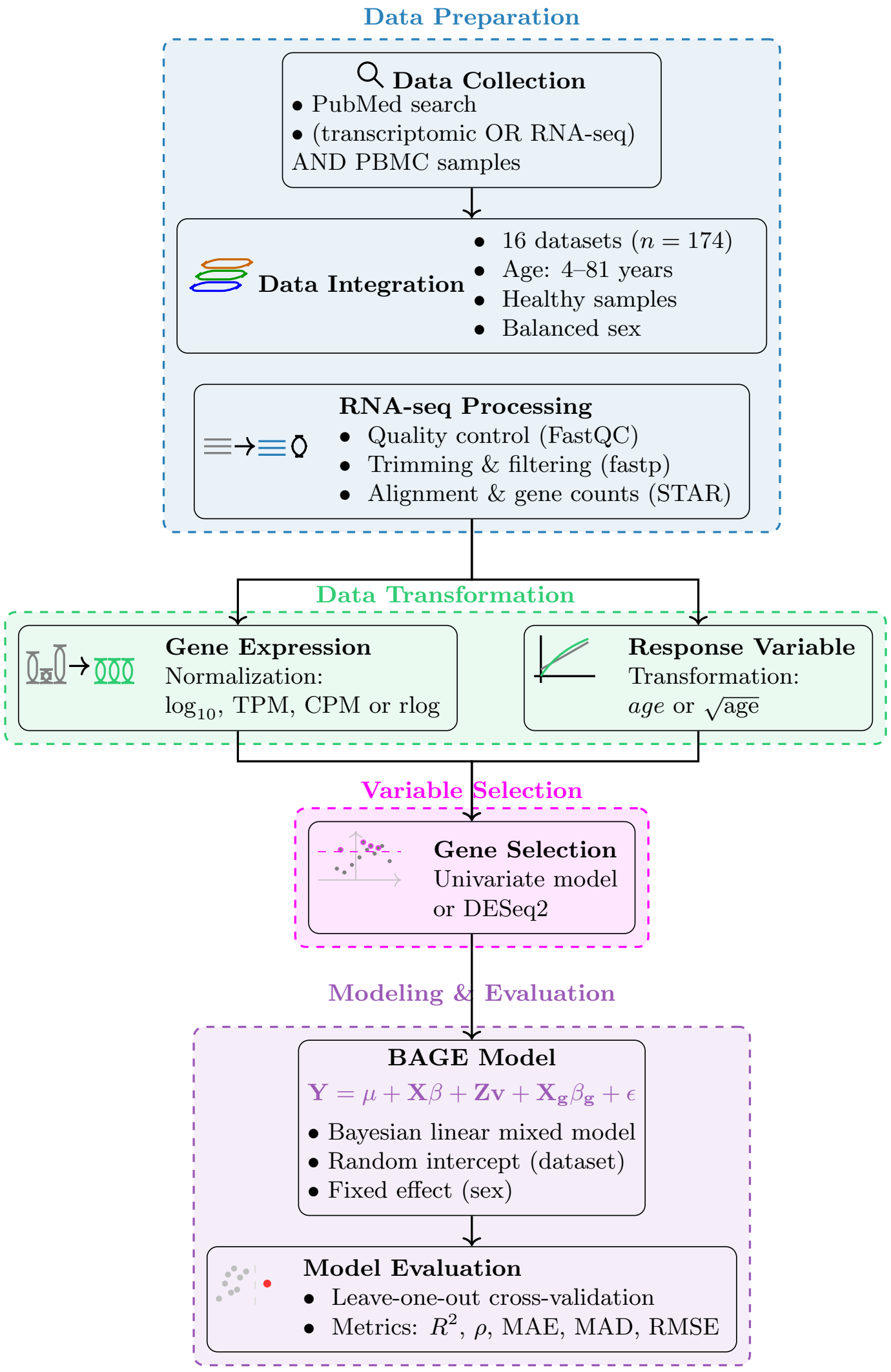

Fig. S1 Schematic overview of the BAGE analytical workflow. The pipeline includes data preparation, transformation of gene expression and response variables, feature selection using statistical approaches, and Bayesian modeling with dataset-specific random effects. Model performance is evaluated using leave-one-out cross-validation across multiple configurations.

Table S1 SRA ID, GEO accession, gender distribution and article of each dataset used in the research.

DATASETS				
SRA ID	GEO	MALE	FEMALE	REFERENCE
SRP281919	GSE159337	4	3	[1]
SRP352989	GSE192829	3	8	[2]
SRP168421	GSE122459	0	6	[3]
SRP341109	NA	1	2	NA
SRP294275	NA	5	3	[4]
SRP072920	GSE79970	0	16	[5]
SRP123453	GSE106443	3	0	[6]
SRP460757	NA	4	4	[7]
SRP327322	GSE179627	17	5	[8]
SRP335222	GSE183204	9	2	[9]
SRP510315	GSE268499	5	0	[10]
SRP473749	GSE248485	6	0	[11]
SRP486949	GSE254647	9	9	[12]
SRP492982	GSE260603	12	20	[13]
SRP473766	GSE248493	10	3	[14]
SRP479103	GSE218999	0	5	[15]

Table S2 Elastic Net performance. Age prediction performance (R^2, ρ, MAE, MAD, RMSE) using an elastic net model for each combination of counts transformation($\log_{10}$, rlog, TPM and CPM) and age transformation indicated. Number of genes correspond to the median of all leave-one-out iterations

Age transformation	# genes	R^2	ρ	MAE	MAD	RMSE
**$\log_{10}$**						
raw	18	0.8046	0.8970	6.4746	4.7174	8.6106
sqrt	22	0.8034	0.8966	6.4070	4.3611	8.6364
**rlog**						
raw	31	0.8005	0.8947	6.7915	5.2615	8.7007
sqrt	40	0.8116	0.9011	6.5470	4.8384	8.4543
**$\log_{10}$(TPM)**						
raw	30	0.7710	0.8784	7.1319	5.9717	9.3204
sqrt	27	0.7788	0.8832	6.9794	5.5584	9.1616
**$\log_{10}$(CPM)**						
raw	31	0.7753	0.8808	7.0846	5.6663	9.2327
sqrt	30	0.7856	0.8867	6.8558	5.3159	9.0191

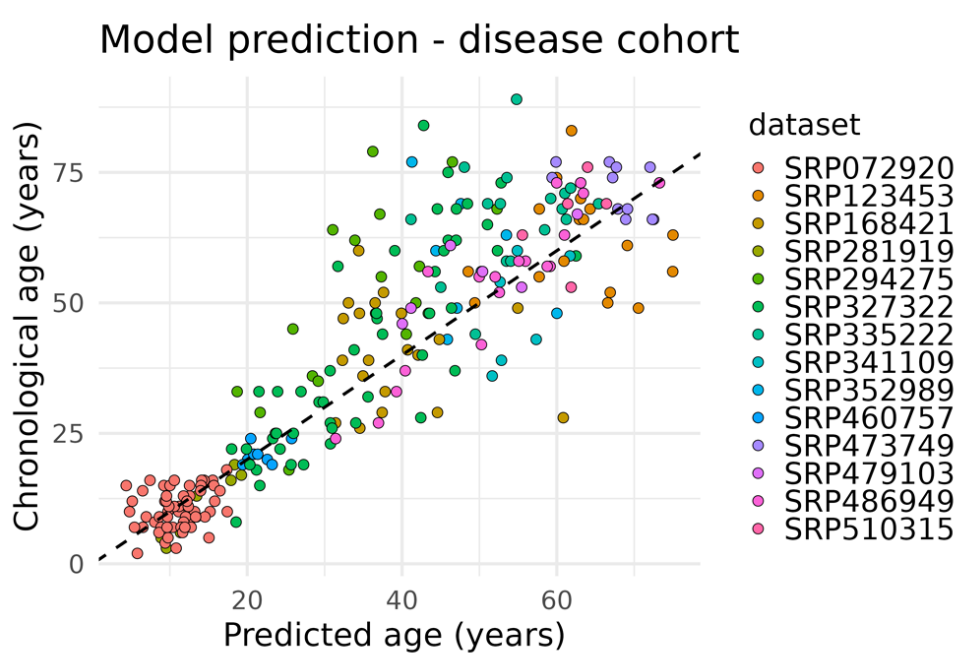

Fig. S2 Model M5 predictions of chronological age on the disease cohort. The plot compares predicted versus chronological age, with each point representing an individual. Points are colored by dataset. The dashed line indicates the 1:1 relationship, where perfect prediction would lie.

Table S3 External validation performance. Age prediction performance (R^2, ρ, MAE, MAD, RMSE) on disease cohort (N=248) using M5 model and BAGE_PBMC signature.

Disease samples				
$\mathbf{R^2}$	ρ	**MAE**	**MAD**	**RMSE**
0.7709	0.8914	8.0689	5.6235	11.3178

Table S4 RNAAgeCalc performance. Age prediction performance (R^2, ρ, MAE, MAD, RMSE) using RNAAgeCalc for each combination of tissue (blood, blood vessel, all_tissues) and signature indicated.

Signature	$\mathbf{R^2}$	ρ	MAE	MAD	RMSE
			**Blood**		
DESeq2	0.2660	0.5186	13.8589	13.4041	16.6889
Pearson	−0.2911	0.4939	18.4716	16.6164	22.1343
Dev	−1.0580	0.2669	23.4414	24.1921	27.9457
deMagalhaes	−0.4474	0.5231	19.9520	19.1336	23.4362
GenAge	−0.0346	0.3971	17.5732	18.0738	19.8145
GTExAge	0.4532	0.7528	12.0533	11.0572	14.4032
Peters	−0.3057	0.8025	18.8257	17.5270	22.2598
all	0.1500	0.5739	14.9128	13.9719	17.9592
			**Blood vessel**		
DESeq2	−0.3330	0.4107	19.2338	18.4513	22.4910
Pearson	−3.8561	0.2425	38.5307	41.0266	42.9268
Dev	−1.4962	−0.1258	26.2440	25.2524	30.7772
deMagalhaes	−10.7707	0.3218	64.0233	63.8552	66.8322
GenAge	−23.1772	0.0466	90.9752	86.7208	95.7828
GTExAge	−0.3123	0.3805	18.1874	15.4862	22.3158
Peters	−0.5086	0.6436	19.9719	19.1360	23.9264
all	0.2133	0.4958	14.4622	13.8455	17.2776
			**All tissues**		
Pearson	−0.9078	−0.0212	22.0671	20.7056	26.9068
Dev	−0.5983	−0.0614	20.9137	20.7306	24.6277
deMagalhaes	−0.57643	0.4539	20.8332	19.8379	24.4583
GenAge	−0.8406	0.1964	22.4446	22.5647	26.4285
GTExAge	−3.2771	−0.1164	34.8935	37.2067	40.2866
Peters	−0.3233	0.4074	18.1585	16.2890	22.4090
all	−1.0732	0.6605	24.3529	26.0809	28.0484

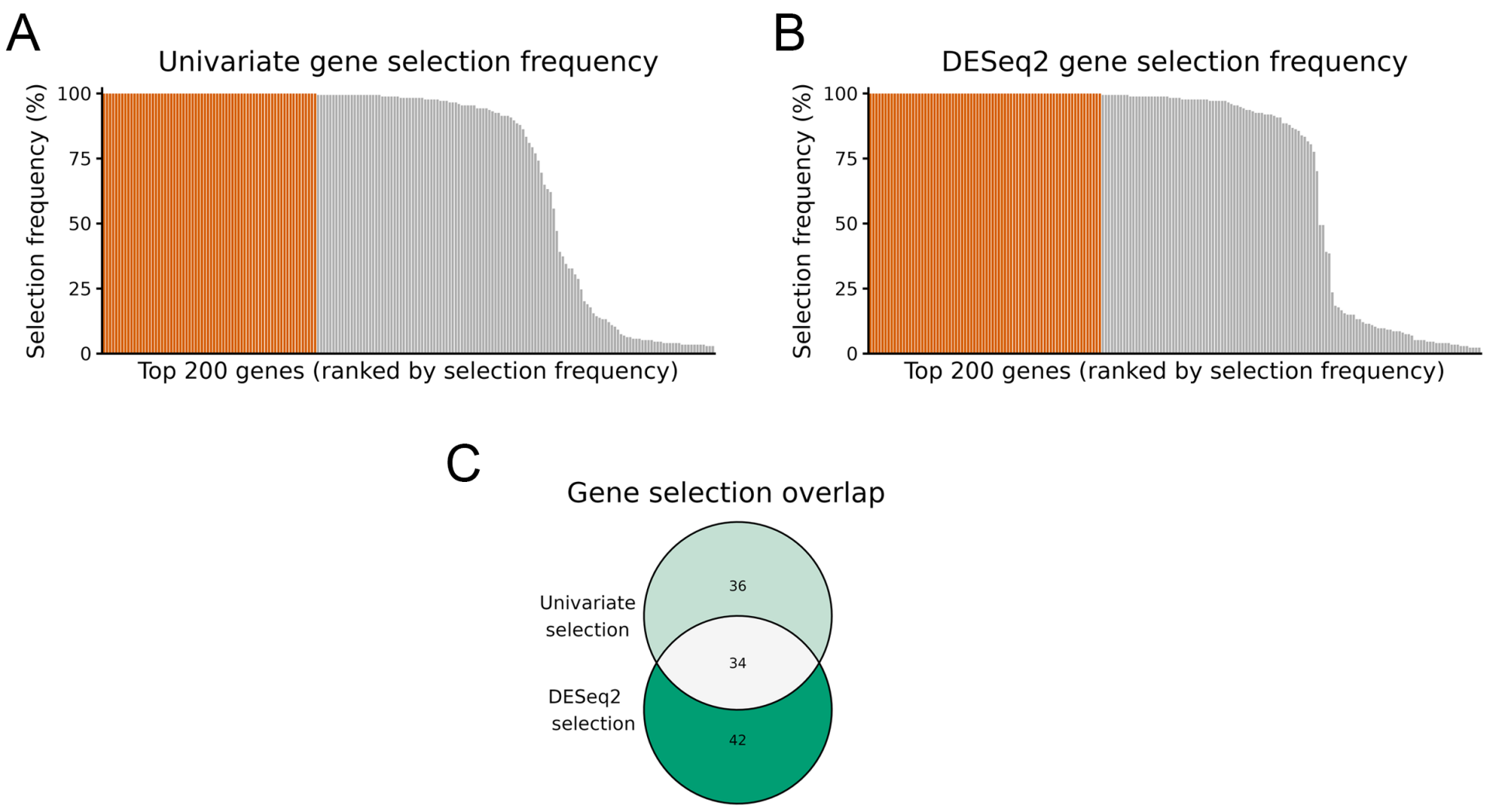

Fig. S3 Variable selection frequency. (A) Frequency of selection variable with univariate linear-model approach. In the x-axis the top 200 genes ranked by number of times selected in the leave-one-out cross validation iterations. In the y-axis we have the percentage indicating the times selected across folds. Orange area indicates the 70 variables (genes) that were selected across all (174) folds, meaning the intersection across folds. (B) Frequency of selection variable with DESeq2 approach, orange area indicates the 76 variables (genes) that were selected across all (174) folds, axis description are the same as in A. (C) Venn diagram of both orange areas in A and B, meaning variables that were selected in 100% of the folds for either univariate or DESeq2 approaches.

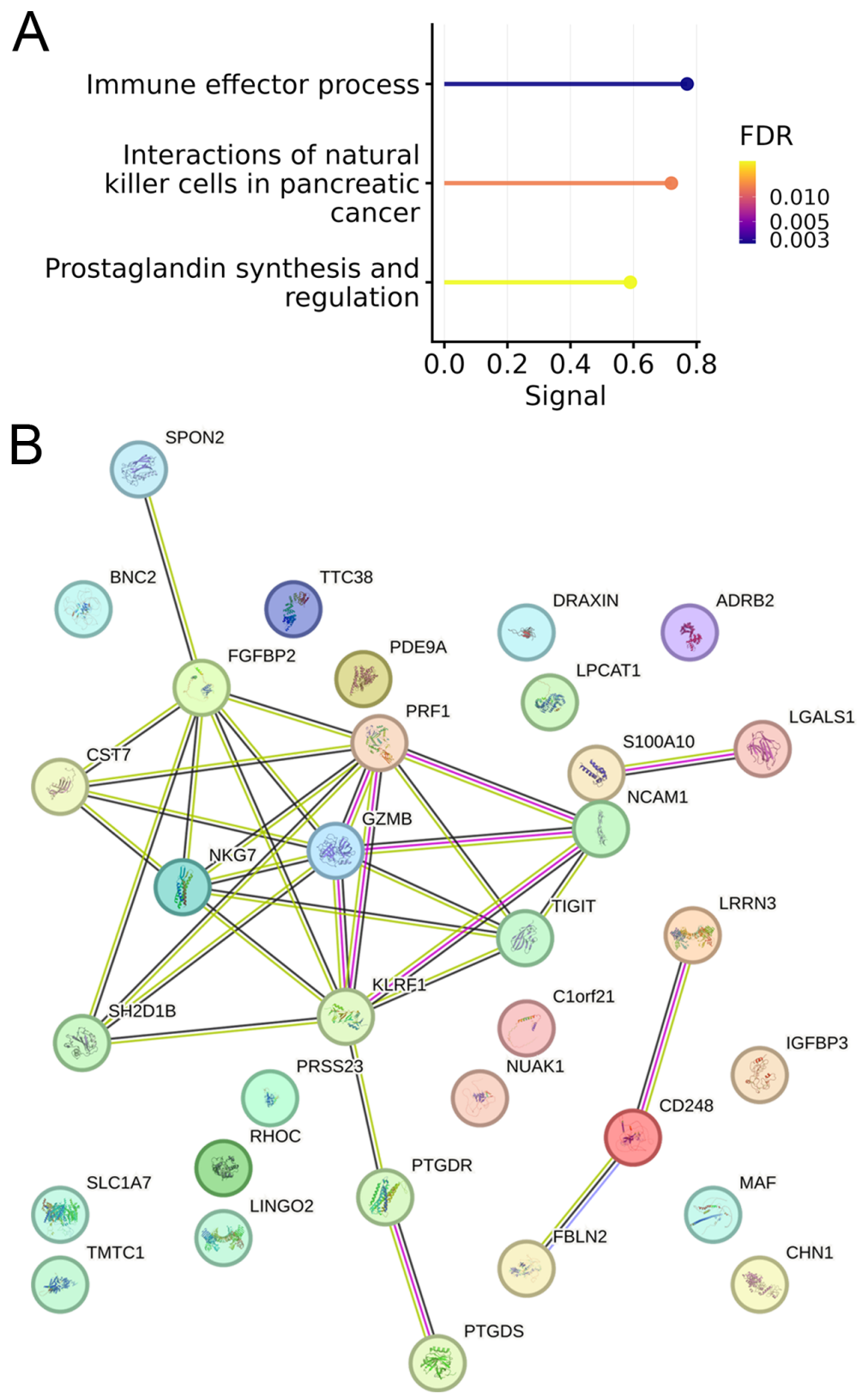

Fig. S4 Gene intersection of Peters' signature with BAGE_PBMC signature. (A) Over-represented pathways with the 32 genes in the intersection of both signatures. (B) Co-association network reconstructed from the 32 genes in the intersection of both signatures.

S2 Methods: Subsampling and bootstraping

After evaluating all models' (M1-M8, D1-D8) performance, we selected the best one in terms of R^2, referred as M5 (see main text, Result section 3.1), and we assessed the stability of the model and of the variable selection procedure under resampling. The assessment had two complementary components:

Subsample stability curve: To quantify how selection and prediction depend on training sample size, we repeatedly fit M5 to random subsamples of the healthy training set (N = 174). For each subsample fraction (50, 60, 70, 80, 90%) we ran 50 independent repeats. We ran these experiments varying the number of selected variables from 50 to 500 in steps of 50. From these runs we recorded model performance and the set of selected predictors to compute retention percentage of the 70 consensus genes.

Bootstrap selection frequency within leave-one-out (LOO) folds: To measure variable selection stability under sampling variability while preserving the leave-one-out cross validation structure, we performed bootstrap resampling inside

each LOO training partition. For each LOO fold k ($k = 1, .., N = 174$) we generated B = 1000 bootstrap samples by sampling with replacement from the fold's training set, and for each bootstrap we applied the same selection pipeline used for M5 and recorded the top 150 ranked genes for that bootstrap replicate. Let $I(g, k, b)$ be an indicator equal to 1 if gene g was selected among the top 150 in bootstrap b of fold k, and 0 otherwise. For each gene g we computed the selection frequency with in each fold, $P(g|k) = \frac{1}{B}\sum_{b=1}^{B} I(g, k, b)$. We then aggregated across folds to obtain an overall selection frequency for each gene, $P(g) = \frac{1}{N}\sum_{k=1}^{N} P(g|k)$. We used these overall frequencies to rank genes by stability and to compare the top 150 ranked stability list against BAGE_PBMC signature (the 70-gene intersection across folds). All bootstrap resampling and selection steps were performed strictly within training partitions to avoid data leakage.

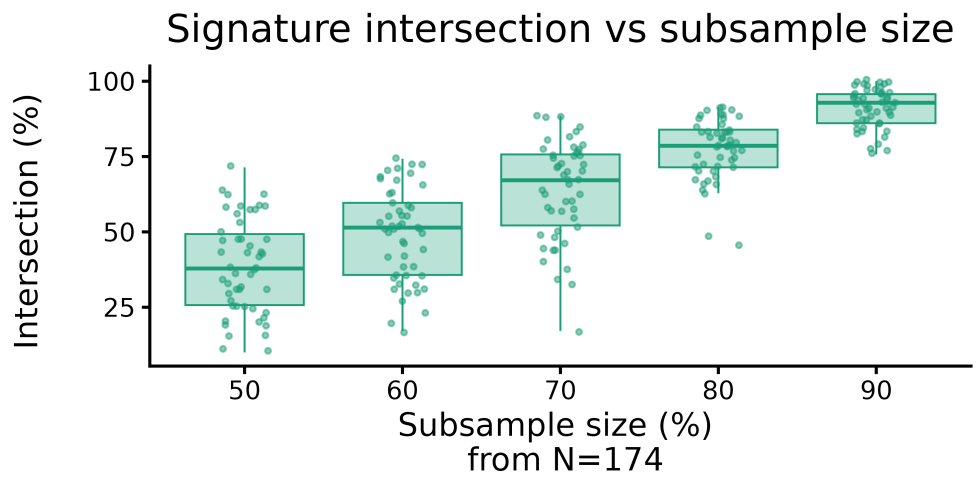

Fig. S5 Gene selection frequency by subsampling and intersection with BAGE_PBMC signature. For each subsample fraction of the original healthy cohort (x axis: 50, 60, 70, 80, 90% of N = 174) we performed 50 independent subsampling runs. In each run we fit model M5, carried out variable selection and recorded the top 150 selected genes. The y axis shows the percentage of genes that overlap between the run's top 150 and the 70-gene BAGE_PBMC signature. Each dot is the overlap observed in a single run. Boxplots summarize the per-fraction distributions (central line = median, bottom edge = first quartile, top edge = third quartile, outliers plotted individually). Higher values indicate greater recovery of the consensus signature at that subsample size.

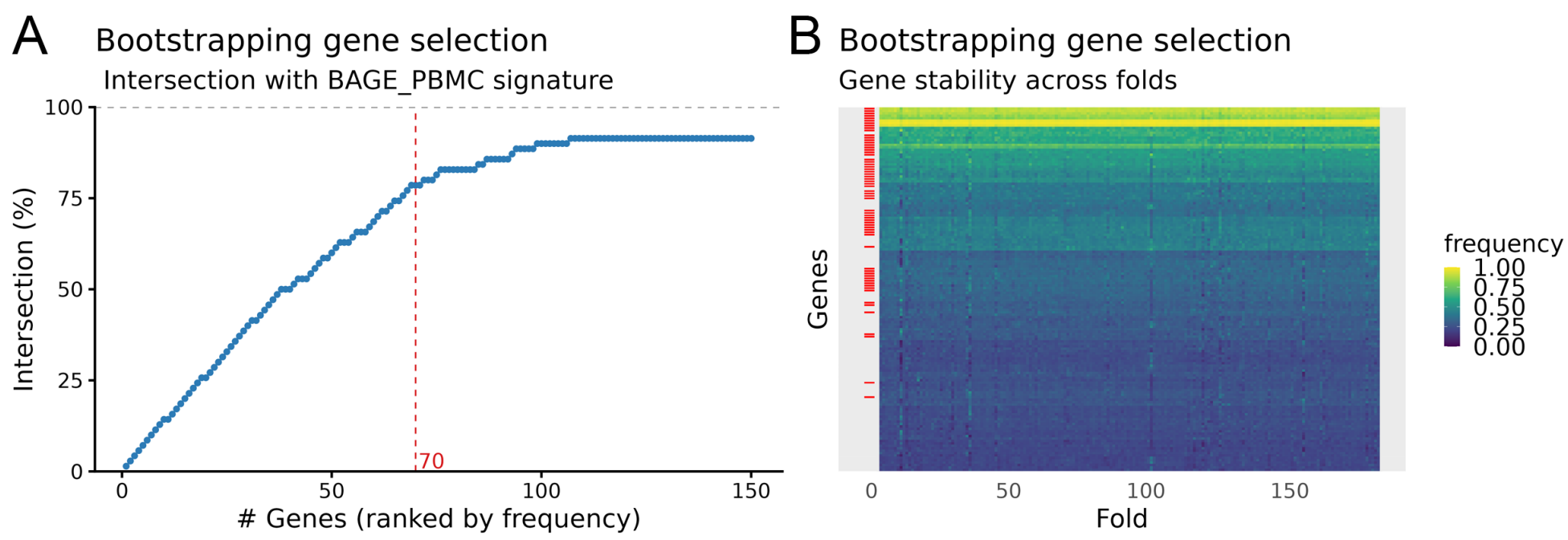

Fig. S6 Gene selection frequency under resampling and its intersection with BAGE_PBMC signature. (A) For each rank cutoff R ($1 \leq R \leq 150$ on the x axis) we formed the set of the R genes with highest overall bootstrap selection frequency P(g), aggregated across folds and bootstraps, and computed the percentage overlap of this top-R set with the 70-gene BAGE_PBMC signature (y axis). The red vertical line indicates $R = 70$; intersects blue line at 78%, under perfect selection stability the top 70 frequent genes would coincide with BAGE_PBMC and the overlap would be 100%. Blue curve plateaus at 91%. (B) Overall gene frequency by fold heatmap. Rows correspond to the genes in the overall top 150 by $P(g)$ (ordered from highest to lowest mean frequency), columns correspond to leave-one-out folds k (174), and each cell displays $P(g|k)$, the fraction of $B = 1000$ bootstrap replicates in fold k in which gene g was selected among the top 150. The color scale runs from 0 (never selected) to 1 (always selected). This panel highlights whether selection of each gene is consistent across different folds. The red annotation on each row represents whether that gene is present in the BAGE_PBMC signature.

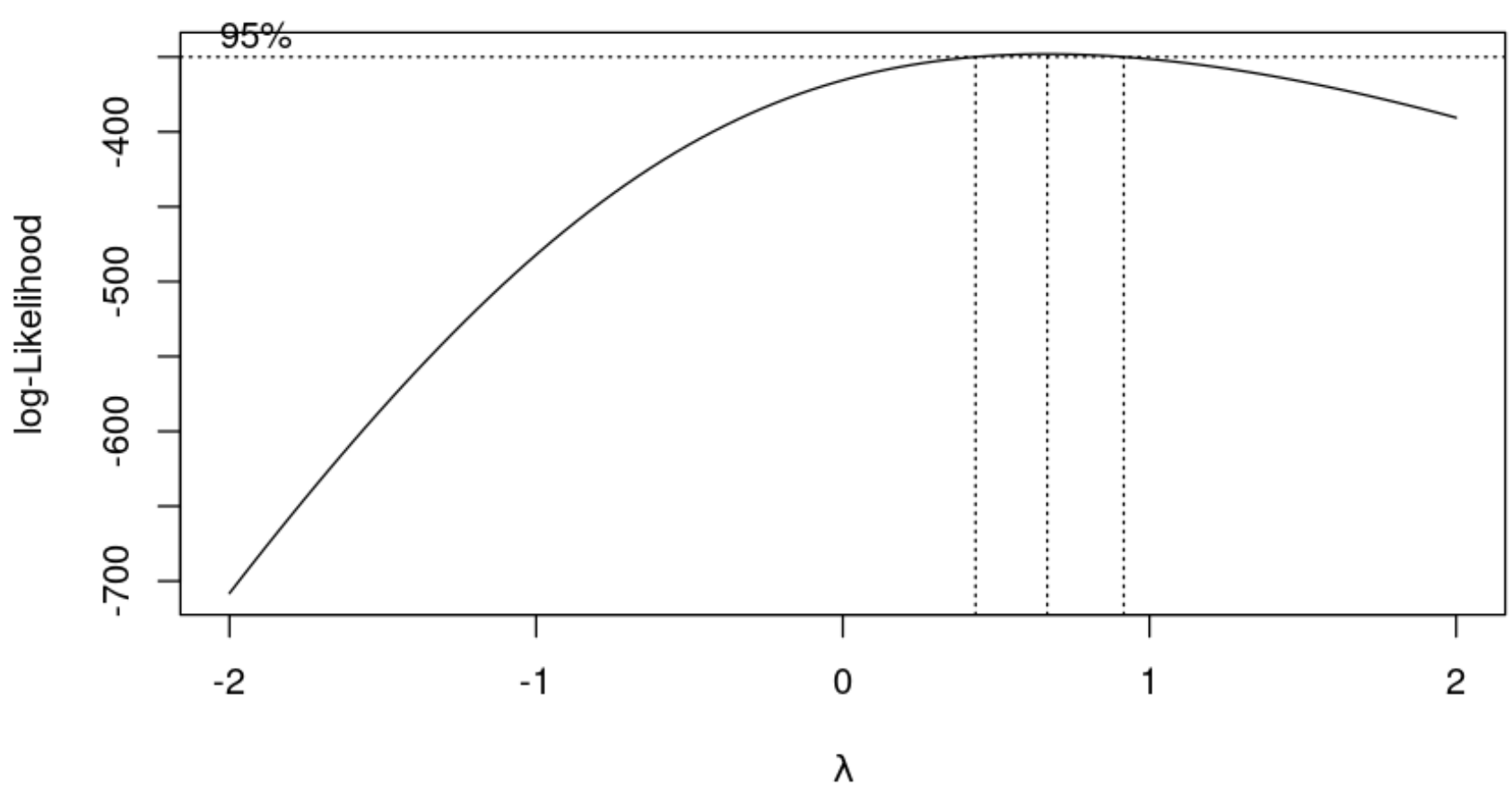

Fig. S7 Grid search for age transformation. Log-likelihoods for the parameter of the Box-Cox power transformation.

References

[1] Huang, Y.-H., Su, T.-C., Wang, C.-H., Wong, S.-L., Chien, Y.-H., Wang, Y.-T., Hwu, W.-L., Lee, N.-C.: Rna-seq of peripheral blood mononuclear cells of congenital generalized lipodystrophy type 2 patients. Scientific data **8**(1), 265 (2021)

[2] Yoshioka, K., Sato, H., Kawasaki, T., Ishii, D., Imamoto, T., Abe, M., Hasegawa, Y., Ohara, O., Tatsumi, K., Suzuki, T.: Transcriptome analysis of peripheral blood mononuclear cells in pulmonary sarcoidosis. Frontiers in Medicine **9**, 822094 (2022)

[3] Tokuyama, M., Kong, Y., Song, E., Jayewickreme, T., Kang, I., Iwasaki, A.: Ervmap analysis reveals genome-wide transcription of human endogenous retroviruses. Proceedings of the National Academy of Sciences **115**(50), 12565–12572 (2018)

[4] Neogi, U., Elaldi, N., Appelberg, S., Ambikan, A., Kennedy, E., Dowall, S., Bagci, B.K., Gupta, S., Rodriguez, J.E., Svensson-Akusjärvi, S., *et al.*: Multi-omics insights into host-viral response and pathogenesis in crimean-congo hemorrhagic fever viruses for novel therapeutic target. Elife **11**, 76071 (2022)

[5] Wong, L., Jiang, K., Chen, Y., Hennon, T., Holmes, L., Wallace, C.A., Jarvis, J.N.: Limits of peripheral blood mononuclear cells for gene expression-based biomarkers in juvenile idiopathic arthritis. Scientific reports **6**(1), 29477 (2016)

[6] Zucca, S., Gagliardi, S., Pandini, C., Diamanti, L., Bordoni, M., Sproviero, D., Arigoni, M., Olivero, M., Pansarasa, O., Ceroni, M., *et al.*: Rna-seq profiling in peripheral blood mononuclear cells of amyotrophic lateral sclerosis patients and controls. Scientific Data **6**(1), 1–8 (2019)

[7] Wei, J., Guo, F., Song, Y., Xu, K., Lin, F., Li, K., Li, B., Qian, Z., Wang, X., Wang, H., *et al.*: Transcriptional analysis of human peripheral blood mononuclear cells stimulated by mycobacterium tuberculosis antigen. Frontiers in Cellular and Infection Microbiology **13**, 1255905 (2023)

[8] Zhang, J., Lin, D., Li, K., Ding, X., Li, L., Liu, Y., Liu, D., Lin, J., Teng, X., Li, Y., *et al.*: Transcriptome analysis of peripheral blood mononuclear cells reveals distinct immune response in asymptomatic and re-detectable positive covid-19 patients. Frontiers in immunology **12**, 716075 (2021)

[9] Garofalo, M., Pandini, C., Bordoni, M., Jacchetti, E., Diamanti, L., Carelli, S., Raimondi, M.T., Sproviero, D., Crippa, V., Carra, S., *et al.*: Rna molecular signature profiling in pbmcs of sporadic als patients: Hsp70 overexpression is associated with nuclear sod1. Cells **11**(2), 293 (2022)

[10] Zhang, J., Xu, S., Liu, J., Liu, T., Fan, Z., Zhou, Y., Basnet, J., Zhang, L., Li,

X., Yang, J., *et al.*: Construction of a cerna network and screening of potential biomarkers and molecular targets in male smokers with chronic obstructive pulmonary disease. Frontiers in Genetics **15**, 1376721 (2024)

[11] Ishii, D., Kawasaki, T., Sato, H., Tatsumi, K., Imamoto, T., Yoshioka, K., Abe, M., Hasegawa, Y., Ohara, O., Suzuki, T.: Effects of anti-fibrotic drugs on transcriptome of peripheral blood mononuclear cells in idiopathic pulmonary fibrosis. International Journal of Molecular Sciences **25**(7), 3750 (2024)

[12] Agrawal, S., Tran, M.T., Jennings, T.S.K., Soliman, M.M.H., Heo, S., Sasson, B., Rahmatpanah, F., Agrawal, A.: Changes in the innate immune response to sars-cov-2 with advancing age in humans. Immunity & Ageing **21**(1), 21 (2024)

[13] Hagenberg, J., Brückl, T.M., Erhart, M., Kopf-Beck, J., Ködel, M., Rehawi, G., Röh-Karamihalev, S., Sauer, S., Yusupov, N., Rex-Haffner, M., *et al.*: Dissecting depression symptoms: Multi-omics clustering uncovers immune-related subgroups and cell-type specific dysregulation. Brain, Behavior, and Immunity **123**, 353–369 (2025)

[14] Imamoto, T., Kawasaki, T., Sato, H., Tatsumi, K., Ishii, D., Yoshioka, K., Hasegawa, Y., Ohara, O., Suzuki, T.: Different transcriptome features of peripheral blood mononuclear cells in non-emphysematous chronic obstructive pulmonary disease. International Journal of Molecular Sciences **25**(1), 66 (2023)

[15] Tao, Y., Xing, S., Zuo, S., Bao, P., Jin, Y., Li, Y., Li, M., Wu, Y., Chen, S., Wang, X., et al.: Cell-free multi-omics analysis reveals potential biomarkers in gastrointestinal cancer patients' blood. Cell Reports Medicine **4**(11) (2023)
